# Does slow release oral morphine have impact on craving and impulsivity in heroin dependent individuals?

**DOI:** 10.1097/YIC.0000000000000418

**Published:** 2022-07-14

**Authors:** Julie Giustiniani, Stéphane Rothen, Louise Penzenstadler, Laura Colombo, Gérard Calzada, Gabriel Thorens, Daniele Zullino

**Affiliations:** a Addictology Division, Mental Health and Psychiatry Department, Geneva University Hospitals; b Research Center for Statistics, University of Geneva, Geneva School of Management and Economics; c Faculty of Medicine, Geneva University, Geneva, Switzerland

**Keywords:** Balloon Analogue Risk Task, craving, delay discounting, impulsivity, slow-release oral morphine, stop-signal reaction time

## Abstract

Craving and impulsivity are addiction components which explain why heroin-dependant individuals (HDI), continue using heroin despite not wanting to do so. Opioid maintenance treatment (OMT), such as slow-release oral morphine (SROM), is the most effective treatment for opioid dependence. However, the impact of SROM on craving and impulsivity remains unclear. In this observational study, 23 HDI receiving SROM, their usual OMT, took part in the experiment. Each of the participants filled in the perceived level of craving with a visual analog scale. Their impulsivity was assessed via three laboratory tasks, the stop-signal reaction time, the Balloon Analogue Risk Task and delay discounting. Each evaluation was performed before and after SROM administration. Craving was significantly reduced after administration of SROM (difference 2.83; *P* = 0.0010), whereas there were no significant differences in performance in the three laboratory tasks. In the long term, we observed an improvement on delay discounting correlated with the duration and dosage of SROM. The acute impact of SROM appears to significantly reduce craving, without impacting impulsivity. Observation of the correlation between delay discounting and the duration and dosage of OMT is of great interest and should be studied further.

## Introduction

Heroin addiction, like other addictions, is a chronic condition characterized by remissions and relapses (Daglish *et al.*, 2001) and a compulsion to seek and use heroin despite negative consequences (Ferri *et al.*, 2011). Indeed, as in other addictions, Heroin dependant individuals (HDI), even if they are aware of the negative consequences and choose to abstain from heroin use, eventually relapse. This loss of control which is one of the main elements of all addictions could be explained by craving and impulsivity (Moshier *et al.*, 2013).

Impulsivity has been described in all addictions and potentially plays a role in all phases of the addiction cycle (Dissabandara *et al.*, 2014; Vassileva *et al.*, 2014). However, impulsivity is a complex construct with several components related to risk-taking and lack of reflection that impact behavior in different ways (Xu *et al.*, 2013; Antons and Brand, 2018). By extension, the term ‘impulsive’ is often used indiscriminately to define a series of maladaptive behaviors that reflect these different components, including the inability to suppress inappropriate behavior (motor impulsivity), risk-taking (risky behaviors) and the inability to defer gratification (impulsive choice) (Bari and Robbins, 2013). Craving, on the other hand, has been identified to occur once an addiction is present and could influence its trajectory. Craving is an irrepressible compulsion to use an addictive substance despite not wanting to do so. Craving could explain persistent substance use (Bernheim and Rangel, 2004) and relapses (Daglish *et al.*, 2001). Thus, craving is a key symptom of substance use disorders (American Psychiatric Association, 2013). Craving can be triggered by cues that, in the context of addiction, may become salient after a process of associative learning. Craving indicates a higher likelihood of an automatic impulsive response to cues rather than a controlled and reflected response (Antons and Brand, 2018). Through this definition, we understand how craving and compulsive behavior are two components that interact and lead to addictive behavior.

HDIs are particularly interesting for studies on impulsivity and craving because they are the only group of patients receiving a single valid treatment, Opioid maintenance treatment (OMT). In addition, they are a specific subpopulation of opioid use disorder (OUD), where the opiate (heroin) is obtained outside a clinical context. The OMT strategy is to substitute an illicit opioid that is particularly addictive due to its shorter action with a licit opioid which can be administered once a day due to its pharmacokinetics (Amato *et al.*, 2013). Indeed, OMT is the most effective treatment for opioid dependence (Carlsen *et al.*, 2020) allowing the reduction or disappearance of withdrawal symptoms and cravings (Fareed *et al.*, 2011). However, its impact on impulsivity and the expression of impulsive behaviors is still unclear. Existing literature showed contradictory data potentially explained by different research methodologies. Indeed, some studies have identified that opioid use, even OMT, leads to higher impulsivity (Odum *et al.*, 2000; Lee and Pau, 2002; Verdejo *et al.*, 2005; Fishbein *et al.*, 2007; Dissabandara *et al.*, 2014; Yang *et al.*, 2015) while others conclude that there is a decrease (Liao *et al.*, 2014; Li *et al*., 2020 or simply no impact (Zacny and de Wit, 2009; Harty *et al.*, 2011). In view of the impact of impulsivity, on the course of addictive disorders, it seems necessary to better understand the impact of OMT on impulsivity. All these data lead us to question whether the long-term prescription of OMT has a favorable or unfavorable impact on the different components of impulsivity and on the disorder itself. To answer this question, we have studied a group of patients undergoing treatment for heroin dependence, all of whom were receiving the same OMT, slow release oral morphine (SROM). SROM has been suggested as an alternative treatment for HDI, especially for those with intolerance for other OMTs (Socias *et al.*, 2020). Although its utilization for opioid dependence is still not authorized in many countries (Koller *et al.*, 2019), it has been available for several years in Switzerland. In this context, the purpose of our study is to determine the impact on craving and impulsivity of a single opioid administration in a therapeutic setting on HDI under OMT with SROM.

## Method

### Study sample

Twenty-three patients were recruited from the Division of Addictology (Service d’Addictologie) of the University Hospital of Geneva (Switzerland) with a diagnostic and statistical manual of mental disorder fifth edition (DSM-5)diagnosis of opioid Dependence. Participants were eligible if they fulfilled all of the following inclusion criteria: informed consent as documented by signature; able to communicate in French; age over 18 years old; on a stable dose of SROM not modified at least 14 days before inclusion. Non-inclusion/exclusion-criteria included unstable psychiatric disorder and acute withdrawal syndrome. Participants received 50CHF-vouchers for their participation. The study was approved by the Ethical Committee of the Canton of Geneva and the Swiss Agency for Therapeutic Products (Swissmedic), and was carried out in accordance to the protocol and according to the principles enunciated in the Declaration of Helsinki and the guidelines of Good Clinical Practice (GCP) issued by International conference on harmonization.

### Assessment measures

#### Craving evaluation

Usually, self-reported general craving for heroin is assessed with three-item Likert-type rating or visual analog scale (VAS). This self-reported measure assesses the global sense of craving. It is easy to use and especially suitable for frequent and repeated measures, it is also sensitive to rapid changes (Yen *et al.*, 2016). In this study, self-reported general craving for heroin was assessed pre- and post-SROM administration using a VAS with three questions: (1) “How would you rate your desire to use in the last 30 min?”; (2) “Imagine yourself in a situation associated with your past substance use; if you were in that place now, how likely would you be to actually use?” and three (3) “Confronted with a triggering situation without the possibility to use immediately, evaluate the intensity of your cravings?”. For every question, subjects could give a score from 0 (not intense at all’) to 10 (‘very intense’) (Falcato *et al.*, 2015). The total score was computed as the sum of the scores for each item.

#### Alcohol, Smoking and Substance Involvement Screening Test

The Alcohol, Smoking and Substance Involvement Screening Test (ASSIST) is a short screening questionnaire developed by the WHO to assess the use of different substances (tobacco products, alcohol, cannabis, cocaine, amphetamine-type stimulants, sedatives and sleeping pills, hallucinogens, inhalants, opiates and ‘other drugs’) and the associated consequences (Humeniuk *et al.*, 2008; WHO ASSIST Working Group, 2002). The ASSIST in its current French version (ASSIST V3.0) [28] is composed of eight questions that determine a risk score for each substance, which allows to conclude the most appropriate intervention for that level of use. The score for each substance is categorized as low risk (occasional or nonharmful use), moderate risk (more regular or harmful use) or high risk (frequent risky use or suggestive of dependence). Each substance has a threshold between each risk category. Scores above 3 for all products (tobacco, cannabis, cocaine, psychostimulants, inhalants, tranquilizers and hallucinogens), with the exception of alcohol (score above 10), are considered moderate or high risk. The ASSIST is therefore a well-validated screening test for substance overuse and dependence in an adult population (Khan *et al.*, 2011).

### Experimental tasks

#### Stop-signal reaction task

The stop-signal reaction task (SSRT) is the prototypical task used to assess the capacity of inhibitory mechanisms, measuring the ability to inhibit an automatic motor response. The task consists in responding to a visual signal (go signal) as fast as possible (go task), but to refrain this answer (stop task) when an auditory signal (stop signal) is heard. The frequency of this stop signal is set on one trial out of four (25%) but the delay between the go signal and this stop signal varies and is successively adjusted to make it tend towards the median reaction time. The latency of the response to the stop signal (stop-signal reaction time) is calculated as a quantitative measure of inhibitory control. Longer stop-signal reaction times are associated with higher impulsivity (Logan *et al.*, 1997).

#### Delay discounting task

The delay discounting task is designed to assess impulsive decision-making (Rachlin *et al.*, 1991). This task uses a computerized adjusting-amount procedure to measure how a delay impinging a granted reward decreases the attractiveness of this reward, hence the term ‘discount’. Hypothetically monetary rewards are adopted to quantify this effect. In a series of choice trials, participants have to decide repeatedly between two options: a smaller amount of money (hypothetically) available immediately or a larger amount of money available after a delay (e.g. $100 immediately or $1000 in 1 year). There are three blocks of trials (1 month, 6 months and 1 year). On each block of trials, the large delayed amount of money is constant across trials, while the immediately available amount of money is changed on each trial. Each of these blocks is presented twice: in one series the immediate smaller amounts of money are presented in descending order and in the other, they are presented in ascending order.

On successive trials, manipulation of parameters allows estimation of the rate of discount, which allows us to find the delay at which the large and the smaller amount of the reward would be valued equally, namely the ‘equivalence point’. The ‘equivalence point’ is calculated by averaging the ascending and descending values for each time period. The ‘equivalence point’ is the value of the last immediate amount when a participant ceases to prefer the immediate amount and chooses the deferred amount, that is the point at which the immediate and deferred amounts have the same subjective value for the participant. In this task, the dependent variable is the area under the curve (AUC) defined by the three equivalence points (1 month, 6 months and 1 year). A lower AUC indicates a stronger preference for smaller sums, but immediate rewards, and is associated with higher impulsivity (Lynam and Miller, 2004).

#### Balloon analogue risk task

The balloon analogue risk task (BART) is a computerized laboratory-based assessment of risk-taking tendencies (Lejuez *et al.*, 2002). In this task, a small, simulated balloon with a balloon pump is displayed on the computer screen.

Participants may inflate the balloon by clicking on the pump in exchange of a monetary reward for each pump. With each click, the balloon inflates and 10 points are added to the participant’s temporary bank. At any point, the participant may decide to stop to inflate the balloon and collect the sum garnered on this balloon. The sum is banked in the permanent bank.

However, each balloon is set to explode at random with the result of the loss of all money accumulated for that balloon. Each balloon has a different explosion point and is programmed to pop between 1 and 64 pumps (maximum number of clicks per balloon). The participants are only informed that the balloon can explode anywhere from the first pump all the way to the point where it fills the whole screen. After each balloon explosion or money collection, a further balloon appears until a total of 30 balloons have spawned.

The main dependent measure on the BART is quantified by the average number of pumps delivered in balloons that did not explode (Lejuez *et al.*, 2002). Higher scores imply a higher risk-taking predilection (Lejuez *et al.*, 2002; White *et al.*, 2008).

### Procedure

This study is an observational study, with a single dose in an open-label design: all subjects received their usual SROM treatment with a stabilized dosage. In the first part of the study, participants had to complete questionnaires about their socio-demographic status and give information about other substances used with the ASSIST self-rating questionnaire. Patients were informed that they would receive their regular dose of SROM during the experiment. In the pre-SROM assessment patients’ craving was evaluated with the instrument described above, and the three experimental tasks (SSRT, delay discounting and BART) were performed. After the pre-SROM assessment patients received their regular dose of SROM. The post-SROM evaluation was performed between 60 and 120 min after drug administration, using the exact same assessments as for the pre-SROM assessment.

### Data analysis

Statistical analyses were conducted using R (R Core Team (2020). Variables were described as frequencies or mean values and SD. To evaluate the acute impact of SROM, the pre/postanalysis of the scores of craving, delay discounting, SSRT and BART for the entire population was performed using the Wilcoxon test for repeated measures. To assess whether the long-term effect of SROM treatment could affect clinical parameters, the relationship between various variables such as craving, positive DSM-5 criteria or number of substances used and overused, and performance obtained in the laboratory task before SROM administration and treatment parameters (SROM duration and dosage) was assessed using nonparametric Spearman rank correlation. All statistical tests were considered significant if *P* < 0.05.

## Results

Sociodemographics, substance use patterns, the severity of the OUD defined by the number of positive DSM-5 criteria, and SROM treatment parameters (duration and dosage) are presented in Table 1. In addition, other substance use was taken into consideration with the ASSIST scores (Table 1).

**Table 1:** Socio-demographic and diagnostic characteristics of the study sample

	Experimental group (n: 23) Mean (SD)	
Age	43.8 (8.6)	
Gender male (%)	20 (87)	
Positive DSM-5 criteria	9.87 (3.9)	
Doses of SROM (mg/day)	528.6 (289.26)	
Duration of SROM treatment (weeks)	128.20 (66.48)	
Total substance used	3.91 (1.38)	
Total substance abused	2.43 (1.04)	
Substance use	Users (%)	Score ASSIST
Tobacco	100	17.65 (5.93)
Alcohol	78	7.56 (8.39)
Cannabis	74	8 (7.66)
Cocaine	65	6.91 (8.66)
Psychostimulant	13	0.39 (1.03)
Hallucinogen	17	0.65 (1.55)
Tranquilizer	43	2.82 (5.37)

DSM-5, diagnostic and statistical manual of mental disorder fifth edition; SROM, slow-release oral morphine.

There was a significant decrease in craving, pre- vs. post-SROM administration with a difference of 2.83 (SD: 1.69), [5.22 (SD: 5.74) vs. 2.39 (SD: 4.05); *P* = 0.0010].

**Fig. 1:**
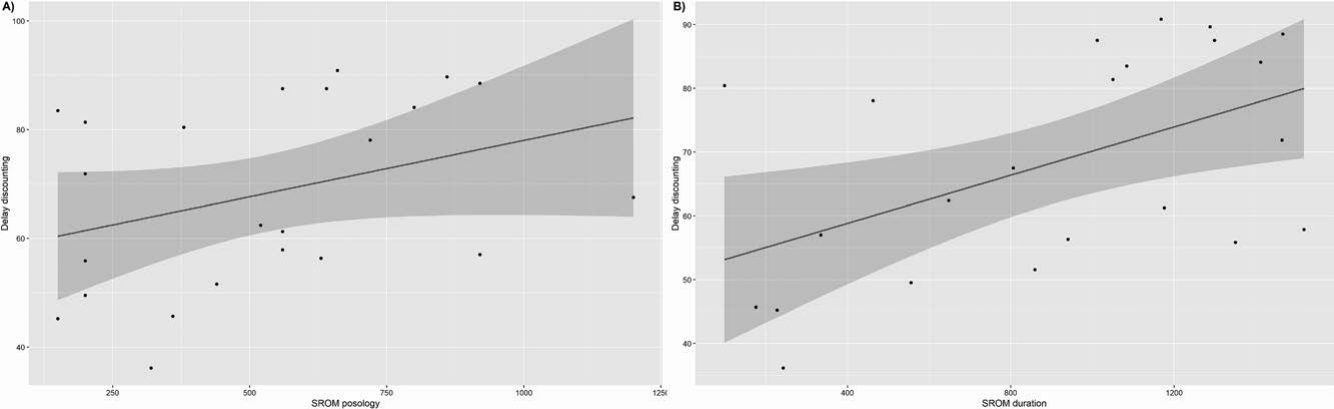
Correlation between the delay discounting task and slow-release oral morphine treatment parameters, A/ dosage in mg/day and B/ duration in weeks.

Further analysis showed a positive correlation between delay discounting and duration of SROM treatment (S = 983.74; *P* value = 0.01212; rho = 0.513961), as well as SROM dosage (S = 1078; *P*-value = 0.02453; rho = 0.4673811) (see Fig. 1). Finally, we found no correlation between SROM treatment parameters (dosage and duration) and perceived craving, other laboratory tasks (SSRT and BART) or even OUD severity.

With respect to the laboratory tasks, we found no correlation between each of them and the number of substances used or overused. However, we found a trend between delay discounting and the number of substances overused (S = 2796.2; *P* value = 0.07243; rho = −0.3815313). This trend is also maintained between delay discounting and the total ASSIST score (S = 2761.7; *P* value = 0.08727; rho = −0.3644906).

In addition, we found no correlation between the number of DSM-5 criteria and treatment parameters or laboratory tasks or the number of substances used/overused.

## Discussion

The analysis showed a positive acute effect of SROM on craving but not on impulsivity, while it’s long-term effect appeared to be inverse with no effect on craving and a positive correlation between SROM treatment parameters and delay discounting.

Indeed, we observed a significant decrease in craving after SROM administration even though SROM dosages were stable for at least 3 months. Concerning the impact on craving, it has been proven for all OMTs that higher dosages are more effective (Fareed *et al.*, 2011). However, higher dosages risk precipitating adverse effects and generating patient resistance in treatment adhesion (Coffa and Snyder, 2019). Thus, SROM appears to be an attractive OMT for patients with cardiac disease or intolerance to other OMTs because of the lesser physical side effects and the lack of impact on the QT interval (Klimas *et al.*, 2019; Verthein *et al.*, 2015). Besides, SROM appears to have similar efficacy on the illicit drug consumption as methadone (Beck *et al.*, 2014) and even superior efficacy for craving (Klimas *et al.*, 2019). Concerning this last point, our data provided some nuances regarding the impact of SROM on craving. Indeed, our data suggest that although SROM is effective on the perception of craving just after administration, it appears that this effect does not persist beyond 24 h as shown by the reappearance of significant craving just before the next treatment intake. Other studies which suggest the superiority of SROM on craving over methadone give no information about when craving was evaluated. These findings on craving appear to be a general perception (Falcato *et al.*, 2015; Klimas *et al.*, 2019). To our knowledge, the pharmacokinetic characteristics of the two molecules could explain why the perception of craving may be different. Methadone, the reference treatment always compared to SROM, is well known for its long elimination half-life of about 24 h (Grissinger, 2011), whereas SROM has a long action due to extended-release capsules with a shorter elimination half-life of about 11–13 h (Gourlay, 1998). From a pharmacokinetic point of view, this SROM characteristic could induce an end-of-dose effect with the re-emergence of more important craving than for methadone. Despite this aspect, it appears that patients report sufficient relief of their craving with SROM (Falcato *et al.*, 2015; Klimas *et al.*, 2019), and a majority even prefer it to methadone (Mitchell *et al.*, 2003). At this stage of our reflection, we can confirm the favorable therapeutic impact of SROM on craving and exclude the risk of dosage escalation.

Regarding acute SROM impact on impulsivity, we did not observe any significate difference in the three tasks before and after SROM administration. This observation is in line with certain previous studies that conclude a lack of impact of opioids on impulsivity (Harty *et al.*, 2011; Zacny and de Wit, 2009). However, these studies were difficult to transpose to alternative clinical settings because their results were derived from animal studies (Harty *et al.*, 2011) or studies on healthy volunteers (Zacny and de Wit, 2009). Studies with opposite results on the impact of opioids on impulsivity found them to either increase or decrease it. These contradictory data seem to be explained by their methodological frameworks that differed significantly. Observation of an increased impulsivity effect of opiates is obtained either by self-report measures (Kirby *et al.*, 1999; Madden *et al.*, 1997; Giordano *et al.*, 2002) or by clinical observation of risky behavior (Odum *et al.*, 2000; Giordano *et al.*, 2002; Dissabandara *et al.*, 2014). Evidence from laboratory tasks that showed a deficit in inhibition and impulse control (Lee and Pau, 2002; Yang *et al.*, 2015; Li *et al*., 2020) and response shifting (Fishbein *et al.*, 2007) induced by opioids, are conducted through population comparison. The methodology applied cannot differentiate the effects of the substance and what may be an inherent component of the addictive disorder. Thus, the increase in impulsivity observed in these studies is much more likely to reflect the addictive disorder than a true pharmacological impact. In fact, in Yang *et al.* (2015), methadone was identified as altering the control of inhibition. However, it seems in this study that the HDI subjects receiving OMT tend to present better performances than the HDI without OMT (Yang *et al.*, 2015). Besides, in a therapeutic context lower impulsivity has been observed in patients who received methadone than in abstinent individuals with a history of heroin dependence (Liao *et al.*, 2014). This observation leads to the hypothesis that opioids used in a therapeutic context could in the long term correct the deficit in inhibitory control observed in HDI (Liao *et al.*, 2014; Yang *et al.*, 2015).

To determine whether the therapeutic context could have a long-term impact on performance we realized a correlation analysis of scores obtained before administration of SROM and the duration of SROM and its dosage. We found a positive correlation between the SROM treatment parameters, such as duration and posology and results obtained in the delay discounting task. These results led us to conclude that the longer the patients have been in treatment and the higher the dosage, the less impulsive they are. This observation between treatment parameters and delay discounting has been made in previous studies with two main possible explanations for this phenomenon. The first explanation was that patients with lower impulsivity are more likely to engage in a long-term care protocol, and show a higher level of adherence to treatment. Indeed, higher impulsivity has been associated with low retention and weak outcomes in psychotherapy (Hershberger *et al.*, 2017). The second explanation is that therapy could have a positive influence on impulsivity and therefore the longer people are treated the less impulsive they would become (Liao *et al.*, 2014; Yang *et al.*, 2015). Only the correlation made on the dosage could lead us to conclude that the molecule may impact on the performances. However, the reality seems more complex. Clinical experiences teach us that subjects who wish to pursue illicit consumption alongside OMT are often reluctant to take higher doses for fear of losing the positive reinforcement of secondary consumption. Thus, a higher dosage could also reflect a higher level of treatment adherence. In our study setting, it is not possible to determine whether less impulsivity in delay discounting is a favorable marker of good adherence to care or whether the quality of treatment improves this aspect.

Our study has several limitations, the main ones being the small sample size and its heterogeneity. Our sample consisted mainly of poly-consumers and only included a very small proportion of women. In fact, among HDI poly-substance use appears to be the rule rather than the exception (Carlsen *et al.*, 2020) and epidemiological studies highlight the under-representation of women (Observatoire européen des drogues et des toxicomanies, 2013; Pierce *et al.*, 2018). Even if these aspects confer certain representativeness of the HDI population, we do not deny the impact on the laboratory performance. Notably, as higher impulsivity in delay discounting has been associated in the previous literature with the severity of addiction (Amlung *et al.*, 2017; Kluwe-Schiavon *et al.*, 2020) and with the number of substances overused (Moody *et al.*, 2016), we performed additional analyses. However, in our study, we failed to identify any correlation between the severity evaluated by the DSM-5 and delay discounting. And in terms of the number of substances overused, we only found a trend with delay discounting, which could be explained by a two-substance threshold effect described previously (Moody *et al.*, 2016), whereas our participants used a mean of 3.91 substances and overused 2.43 substances. Another limitation is our lack of knowledge of the last substance used, whose acute effect may affect the performance in the tasks. Finally, the last limitations were caused by the open-label observational design. Thus, to conclude on the acute and long-term effect, respectively, two different designs should be considered. Regarding the acute effect of SROM, it will be interesting to continue the investigation with a double-blind randomized controlled trial to compare each opioid. Indeed, the current methodology does not allow concluding on a specific action of SROM. Considering the result of the delay discounting, it will be very interesting for future research to explore this aspect in a longitudinal protocol, to clarify whether the better performance at delay discounting reflects a better prognostic or a positive impact of the therapeutic.

In conclusion, we were able to demonstrate the acute therapeutic impact of SROM on craving, with no impact on impulsivity. Finally, the observation of the correlation between delay discounting and the duration and dosage of OMT is of great interest and should be studied further.

**Table 2:** Effect of slow-release oral morphine administration

Measurements	Before SROM administration	After SROM administration	*P* value	Effect size
Craving	5.22 (SD: 5.74)	2.39 (SD: 4.05)	0.0010	0.759
Impulsivity measures				
BART^a^	16.28 (SD: 6.57)	17.49 (SD: 8.18)	0.211	0.266
SSRT^b^	323.53 (SD: 43.66)	330.56 (SD: 77.79)	0.823	0.050
Delay discounting^c^	68.24 (SD: 16.98)	69.10 (SD: 18.15)	0.961	0.006

BART, Balloon Analogue Risk Task; SROM, slow-release oral morphine; SSRT,stop-signal reaction time.

a Mean adjusted pumps.

b Mean reaction time in ms.

c AUC.

## Acknowledgements

The authors thank all volunteers for participating in the study.

This study was supported by a grant from Mundipharma Medical Company and CARIGEST SA: www.carigest.ch (24, rue de l’Athénée, CH - 1206 Genève, +41 (0)22 839 72 90 Fax: +41 (0)22 839 72 99, carigest@carigest.ch.

Conceived and designed the experiments: D.Z., G.T., S.R., G.C. Performed the experiments: G.C. Analyzed the data: L.C. and J.G. Contributed reagents/materials/analysis tools: L.C., S.T. and J.G. Contributed to the writing of the manuscript: J.G., G.T., S.R., G.C., L.P., D.Z.

### Conflicts of interest

G.T. has received reimbursement for attending congresses from the following companies: Eli Lilly; D.Z. has received research support from Eli Lilly, Organon, Wyeth, Sanofi-Synthelabo, Aventis and Janssen-Cilag; He is/has been a member of advisory boards for Eli Lilly, Wyeth, Astra Zeneca, Pfizer and Lundbeck; He has received speakers fees from Astra Zeneca, Eli Lilly, Janssen-Cilag, GlaxoSmithKline, Novartis, Pfizer, Organon, Wyeth, Lundbeck. He has received reimbursement for attending congresses from the following companies: Eli Lilly, Wyeth, Astra Zeneca, GlaxoSmithKline, Organon, Janssen-Cilag, GlaxoSmithKline, Lundbeck and Mundipharma. For the remaining authors, there are no conflicts of interest.
